# Developmental and functional relationships between hypothalamic tanycytes and embryonic radial glia

**DOI:** 10.3389/fnins.2022.1129414

**Published:** 2023-01-20

**Authors:** Harmony Fong, Deborah M. Kurrasch

**Affiliations:** ^1^Department of Medical Genetics, Cumming School of Medicine, University of Calgary, Calgary, AB, Canada; ^2^Alberta Children’s Hospital Research Institute, University of Calgary, Calgary, AB, Canada; ^3^Hotchkiss Brain Institute, University of Calgary, Calgary, AB, Canada

**Keywords:** tanycyte, radial glia, neural stem cell, neurodevelopment, hypothalamus

## Abstract

The hypothalamus is a key regulator of several homeostatic processes, such as circadian rhythms, energy balance, thirst, and thermoregulation. Recently, the hypothalamic third ventricle has emerged as a site of postnatal neurogenesis and gliogenesis. This hypothalamic neural stem potential resides in a heterogeneous population of cells known as tanycytes, which, not unlike radial glia, line the floor and ventrolateral walls of the third ventricle and extend a long process into the hypothalamic parenchyma. Here, we will review historical and recent data regarding tanycyte biology across the lifespan, focusing on the developmental emergence of these diverse cells from embryonic radial glia and their eventual role contributing to a fascinating, but relatively poorly characterized, adult neural stem cell niche.

## Introduction

The hypothalamus is a small but powerful brain region that functions as the body’s homeostatic control center, regulating fundamental processes such as feeding, reproduction, and sleep, as well as social behaviors including parenting and attachment (Saper and Lowell, 2014). Corresponding with its exquisite functional diversity, the hypothalamus contains a vast array of unique neurons and glia, which populate its anterior, tuberal, and mammillary regions along the rostrocaudal axis. Intriguingly, while the subventricular zone of the lateral ventricles and the subgranular zone of the hippocampus are the best-known neural stem cell niches in the adult rodent brain, the hypothalamic ventricular zone is increasingly being recognized as a third site of postnatal neurogenesis and gliogenesis (Goodman and Hajihosseini, 2015; Yoo and Blackshaw, 2018). These newborn neurons and/or glia in the hypothalamus could facilitate plasticity in the circuits controlling various physiologies and behaviors.

Postnatal hypothalamic neural stem potential is largely thought to reside in tanycytes (coined from the Greek word *tanus*, meaning “elongated”), an apparently multifunctional cell population that can contribute to diverse physiological processes (Horstmann, 1954; Goodman and Hajihosseini, 2015; Prevot et al., 2018; Yoo and Blackshaw, 2018). In this review, we provide an overview of tanycyte biology from the developing to the mature hypothalamic niche.

## Morphology, localization, and molecular features of tanycytes

In the adult brain, tanycytes resemble embryonic radial glia not only in function, but also in morphology, location, and molecular profile. Tanycytes position their cell bodies along the walls of the third ventricle and are characterized by a long basal process that projects into the hypothalamic parenchyma or toward portal vessels in the median eminence (Rodriguez et al., 2005). Together with multi-ciliated cuboidal ependymal cells, tanycytes form a tight boundary between the ventricular cerebrospinal fluid, which directly contacts the cells’ uni- or bi-ciliated apical surface, and the flanking parenchyma (Mirzadeh et al., 2017). Along the rostrocaudal axis, tanycytes are abundant within the tuberal hypothalamus, particularly around the level of the median eminence (Millhouse, 1971; Rodriguez et al., 2005; Mathew, 2008). In addition, tanycytes are found along most of the dorsoventral length of the hypothalamic third ventricle, comprising the majority cell population of the floor and ventrolateral walls, then progressively decreasing in density dorsally; following a transition zone of interdigitating tanycytes and ependymal cells, the dorsal-most aspect of the third ventricle is lined almost exclusively by ependymal cells (Millhouse, 1971; Mathew, 2008). With respect to gene expression, tanycytes in seemingly all mammalian species broadly express numerous hypothalamic progenitor and/or adult neural stem cell markers (Pellegrino et al., 2018), including a variety of transcription factors (Rax, Lhx2, Sox2, Sox9), intermediate filaments (Nestin, Vimentin), and Notch signaling components (Notch1, Hes5), among others (Lee et al., 2012; Miranda-Angulo et al., 2014; Salvatierra et al., 2014).

## Development of tanycytes

From a developmental view, the retention of radial glial morphology and at least some neurogenic and/or gliogenic function in tanycytes is particularly interesting, raising the question as to when and how tanycytes differentiate from embryonic radial glia. While the development of tanycytes is poorly characterized compared to that of other hypothalamic cell types, there is a growing body of data that refines the current understanding of tanycyte ontogeny.

Birthdating studies using ^3^H-thymidine autoradiography in the rat hypothalamus indicate that cells lining the third ventricle emerge during late embryonic development. Multi-ciliated ependymal cells, which are thought to possess minimal, if any, neural stem capacity in the mature hypothalamus (Robins et al., 2013), were reported to be generated before tanycytes, with the bulk of rat ependymogenesis occurring between embryonic day (E) 16 and E20 and peaking at approximately E18 (Altman and Bayer, 1978; Das, 1979). Tanycyte differentiation, on the other hand, was observed to start around E19 (equivalent to E17 in mice) and continued through the first and second postnatal weeks (Altman and Bayer, 1978; Das, 1979). These early investigations conclude that tanycytes terminally differentiate from the same radial glial cells that, just a few days earlier, gave rise to neurons of the ventromedial and dorsomedial hypothalamic nuclei. In contrast, however, a recent study employing single-cell RNA sequencing (scRNA-seq) of Rax^+^ lineage cells and EdU birthdating in mice suggests that a subset of radial glia exit the cell cycle to become primitive tanycytes as early as E13, with the majority of tanycytes born between E13 and E15 (Zhang et al., 2021). Species differences and/or differences in the duration of the birthdating chase periods may account, at least in part, for these disparate results; however, it appears that tanycytes may be specified much earlier than previously thought and indicates that tanycytes are formed in parallel with—rather than after—neurons (Shimada and Nakamura, 1973) and other glia in the developing hypothalamus (Marsters et al., 2016). These findings also provide evidence for a “state-switching” model for embryonic hypothalamic progenitors (Zhang et al., 2021). Indeed, results from a genetic inducible fate mapping experiment in the developing mouse hypothalamus are consistent with this inference, with Shh-expressing progenitors labeled before E9.5 contributing neurons and astrocytes to the posterior tuberal and mammillary regions, in addition to generating tanycytes at the third ventricle floor, near the median eminence (Alvarez-Bolado et al., 2012).

Importantly, the developmental programs that govern the formation of tanycytes from embryonic hypothalamic progenitors are now being revealed. With respect to the intrinsic mechanisms that direct tanycyte development, recent transcriptional profiling of the hypothalamus across a comprehensive range of embryonic and postnatal timespoints provides valuable insights into the combinatorial code of regulons, or transcription factor-target gene regulatory networks, that shape hypothalamic cell type identity (Kim et al., 2020; Romanov et al., 2020; Zhang et al., 2021). Prospective glia in the hypothalamus show high activity of the *Hes5*, *Sox9*, and *Nfia* regulons, and within this lineage, tanycytes are further specified by *Nr1d1*; notably, *Nfia*-knockout mice exhibit impaired formation of tanycytes and astrocytes at E18.5 (Romanov et al., 2020). In addition, during the early stages of tanycyte and ependymal cell development, a variety of transcription factors such as *Nr2f1* and *Nfib* are differentially expressed between the two cell types, and are thus candidates to drive ventricular lineage specification (Kim et al., 2020). Consistent with an earlier time-of-origin for tanycytes, this developmental bifurcation between tanycytes and ependymal cells may occur as early as E13 in mice, the timespoints at which differential expression of *Rax* and *Foxj1*—well-established transcriptional regulators of tanycyte and ependymal cell fate, respectively—is first detected (Jacquet et al., 2009; Miranda-Angulo et al., 2014; Kim et al., 2020). In mice with *Rax* haploinsufficiency, ventral expansion of the ependymal cell marker *Rarres2* is observed, and selective knockout of *Rax* in early hypothalamic progenitors leads to a loss of tanycyte-specific gene expression in the third ventricle wall (Miranda-Angulo et al., 2014; Salvatierra et al., 2014). Both effects are phenocopied by the deletion of *Lhx2*, the upstream activator of Rax, from tuberal hypothalamic progenitors, with presumptive tanycytes along the ventral aspect of the third ventricle also exhibiting a hybrid tanycyte-ependymal cell identity (Salvatierra et al., 2014). Indeed, these hybrid cells maintain a radial glia-like morphology while becoming multi-ciliated, suggesting that Lhx2 not only promotes tanycyte differentiation, but also represses ependymal cell fate in the developing hypothalamus (Salvatierra et al., 2014).

Much less is currently known about the extrinsic factors that influence the formation of tanycytes. scRNA-seq data suggests that various morphogenic and growth factor signaling components such as *Wnt7b*, *Ptch1*, and *Igfbp2* are enriched in early stages of their developmental trajectory (Kim et al., 2020), and a knockout mouse model indicates that at later timespoints, the cell adhesion molecule NrCAM plays a role in regulating postnatal tanycyte number (Moore et al., 2022). In addition, microglia may modulate tanycyte development, as these phagocytic cells have emerged as key modulators of diverse neurodevelopmental processes in many areas of the central nervous system, including the hypothalamus (Bilimoria and Stevens, 2015; Rosin and Kurrasch, 2021). In the developing hypothalamus, microglia influence gliogenesis and oligodendrocyte precursor cell migration from the ventricular zone (Marsters et al., 2020), and further, a subpopulation of stress-responsive microglia lie adjacent to and influence neural stem cells along the embryonic third ventricle (Rosin et al., 2021). These effects, likely mediated through secreted cytokines, together raise the possibility that similar extrinsic mechanisms contribute to the development of ventricle-residing tanycytes (Marsters et al., 2020; Rosin et al., 2021).

Following their specification, tanycytes are thought to mature over a protracted postnatal period, reaching terminal differentiation in rodents by approximately 4 weeks after birth according to cytological, histochemical, and ultrastructural criteria (Rodriguez et al., 2005). The apical profile of postnatal day (P) 0 mouse tanycytes, for example, resembles that of embryonic radial glia, and maturation of the apical surface occurs over the first month of life (Mirzadeh et al., 2017). Furthermore, Golgi analyses of the rat hypothalamus reveal that P5 tanycyte processes tend to be shorter and devoid of fine spines when compared to those at P60 (Altman and Bayer, 1978). Between P0 and P10 in mice, cells along the ventrolateral walls of the third ventricle demonstrate gradual downregulation of the radial glia marker RC2, and a concomitant upregulation of GFAP and GLUT1, consistent with a progressive generation of tanycytes from radial glia (de Vitry et al., 1981; Silva-Alvarez et al., 2005; Mirzadeh et al., 2017). These histochemical changes also are accompanied at the ultrastructural level by increases in neuroglial contacts, organelle content, and the number and size of lipid bodies, as determined through electron microscopy of tanycytes in the median eminence of E18 to P7 rats (Rutzel and Schiebler, 1980).

Interestingly, tanycyte development across both embryonic and postnatal stages displays regional variation, suggesting unappreciated heterogeneity in this process. According to incidental observations from early birthdating studies, tanycytes located at the floor of the hypothalamic third ventricle are generated before those occupying more dorsal regions (Altman and Bayer, 1978). Additionally, tanycytes around the level of the arcuate nucleus acquire adult fine structure and potentially function as early as the first postnatal week, while those near the ventromedial nucleus need more time to mature and may not be operative in the early postnatal period (Walsh et al., 1978). Given these observations, it is likely that at the molecular level, tanycyte developmental regulons are differentially activated as a function of dorsoventral positioning along the third ventricle, but this remains to be determined.

## Tanycyte subtypes and heterogeneity

Although the developmental timing and mechanisms of diversification are still unclear, tanycytes in the mature hypothalamus are a distinctly heterogeneous population of cells. This heterogeneity was recognized even in initial characterizations of the cells using the Golgi method, which proposed that tanycytes “may not be identical cytochemical units” (Millhouse, 1971). In the 1970s, a series of enzyme histochemical studies on rat tanycytes was conducted under physiological and experimental conditions, and proved to be immensely influential, establishing a four subtype classification that prevails today. Both deafferentation of the medial basal hypothalamus and bilateral adrenalectomy led to changes in tanycyte metabolic activity that varied with localization along the third ventricle (Akmayev et al., 1973; Akmayev and Fidelina, 1974). Moreover, only a subpopulation of ventrally located tanycytes showed sex differences in metabolic activity during the critical perinatal period of hypothalamic sexual differentiation (Akmayev and Fidelina, 1976). Based on these collective observations, tanycytes were divided into four subtypes—α1, α2, β1, and β2—according to dorsoventral position (Akmayev et al., 1973; Akmayev and Fidelina, 1974, 1976). Subsequent ultrastructural analyses adopted and corroborated this nomenclature, describing differences in fine structural features such as lipid inclusions, endocytic machinery, and spines between the tanycyte subtypes (Rodriguez et al., 1979, 2005, 2019).

**FIGURE 1:**
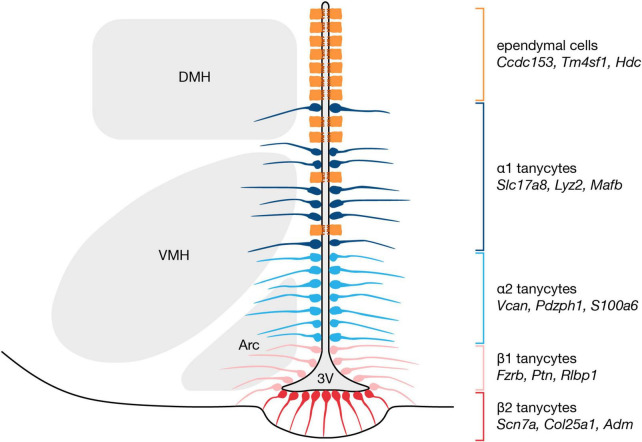
Organization and markers of tanycytes subtypes along the hypothalamic third ventricle. Tanycytes are classified into four subtypes according to dorsoventral location, and recent scRNA-seq studies have identified marker genes for each subtype (Campbell et al., 2017; Chen et al., 2017; Sullivan et al., 2022). α1 tanycytes are the most dorsally located subtype, followed by α2 tanycytes, then β1 tanycytes, and finally β2 tanycytes which occupy the ventricular floor. 3V, third ventricle; Arc, arcuate nucleus; DMH, dorsomedial hypothalamic nucleus; VMH, ventromedial hypothalamic nucleus.

In the tuberal hypothalamus, from dorsal to ventral, α1 tanycytes face the dorsomedial and ventromedial nuclei, α2 tanycytes border the arcuate nucleus, β1 tanycytes occupy the lateral extensions of the third ventricle, and β2 tanycytes reside at the third ventricle floor (Figure 1; Rodriguez et al., 2005). More recently, the four classical tanycyte subtypes have been delineated based on gene expression profiles. From cross-referencing hypothalamic scRNA-seq data with *in situ* hybridization data form the Allen Mouse Brain Atlas, potential markers for α1 tanycytes include *Slc17a8* and *Lyz2*, and α2 tanycytes may be characterized by *Vcan* and *Pdzph1* expression (Campbell et al., 2017; Chen et al., 2017). Likewise, possible marker genes for β1 and β2 tanycytes include *Frzb* and *Scn7a*, respectively (Campbell et al., 2017; Chen et al., 2017). Neural stem markers also are differentially expressed across tanycyte subtypes, which may reflect diverse neurogenic and/or gliogenic potential, and intriguingly, some appear specific to small subpopulations of a given subtype. For example, while GLAST expression distinguishes α tanycytes from β tanycytes, the α tanycyte population itself can be further subdivided into discrete GFAP-expressing dorsal and Prss56-expressing ventral subsets (Robins et al., 2013; Jourdon et al., 2016). In addition, the expression of many tanycyte-enriched genes—neural stem-related or otherwise—is graded with dorsoventral positioning along the third ventricle (Goodman and Hajihosseini, 2015; Campbell et al., 2017; Chen et al., 2017), further complicating the assignment of distinct transcriptomic profiles to each subtype. These expression gradients may reflect the presence of transition zones between tanycyte subtypes, or alternatively, might suggest that tanycytes exist along a continuum of phenotypes (Mathew, 2008; Goodman and Hajihosseini, 2015).

Taken altogether, it is possible, and perhaps even likely, that the traditional four subtype classification does not fully capture the molecular heterogeneity displayed by tanycytes. Sub-clustering of non-neuronal cells from an arcuate nucleus and median eminence scRNA-seq dataset, for instance, identified subclasses of α1 and β2 tanycytes, yielding a total of six potential tanycyte subtypes (Campbell et al., 2017). In addition, *Sprr1a* transcripts were detected in a small number of cells assigned to either the β1 or β2 tanycyte subtypes, and Sprr1a immunoreactivity was precisely restricted to the lateral aspects of the third ventricle floor (Campbell et al., 2017). As such, *Sprr1a* expression may characterize an additional tanycyte subtype occupying the border between the arcuate nucleus and the median eminence, a region that is traditionally regarded as part of the β1 tanycyte domain. Further molecular profiling is likely to uncover other new tanycyte subtypes along the hypothalamic third ventricle, and may also provide insight into an apparently related cell type that resides in the median eminence proper; due to shared ultrastructure, gene expression, and elongated morphology with ventricular tanycytes, some have referred to these cells as γ tanycytes (Wittmann et al., 2017; Wittmann and Lechan, 2018), though others designate them as pituicytes (Rodriguez et al., 2019).

Given the puzzling and seemingly increasing heterogeneity of tanycytes, alternative criteria have been proposed to facilitate categorization of these cells. One recently advanced classification system groups tanycytes according to the nature of the blood vessels and the neuroendocrine axons that their basal process are associated with, and distinguishes four subpopulations of tanycytes: dorsomedial and ventromedial nuclei, dorsomedial arcuate nucleus, ventromedial arcuate nucleus, and median eminence tanycytes (Prevot et al., 2018). These four classes are largely congruent with the classical α1, α2, β1, and β2 subtypes, but this approach may provide a more robust framework for classifying any newly identified tanycyte phenotypes. At variance, cataloging tanycytes based on apical organization is somewhat inconsistent with the traditional tanycyte classification (Mirzadeh et al., 2017). Morphological, ultrastructural, and immunohistochemical characterization of the third ventricle apical surface identified three cell types, termed E1, E2, and E3 cells, that correspond to ependymal cells, α tanycytes, and β tanycytes, respectively (Mirzadeh et al., 2017). In the caudal direction, the E3 domain expands dorsally at the expense of the E2 domain, but the strictly dorsoventral assignment of the classical subtypes implies that α and β tanycytes maintain a uniform distribution along the rostrocaudal axis (Mirzadeh et al., 2017). Altogether, further refinements of tanycyte classification are likely needed to better delineate the heterogeneity of these cells, and to more reliably identify tanycyte subtypes that together comprise a complex neural stem cell niche.

## Neural stem potential of postnatal tanycytes

Apart from the fascinating range of homeostatic processes that tanycytes have been implicated to participate in, which has been discussed in many excellent reviews (Rodriguez et al., 2005; Bolborea and Dale, 2013; Ebling, 2015; Goodman and Hajihosseini, 2015; Prevot et al., 2018; Bolborea and Langlet, 2021), perhaps one of the most perplexing functions of tanycytes is their neurogenic and gliogenic potential in the postnatal hypothalamus. This has been explored in greater detail elsewhere (Yoo and Blackshaw, 2018; Sharif et al., 2021), but in brief, tanycyte neural stem capacity appears heterogeneous with respect to time and the types of neural cells that are generated. Shortly after birth, Nestin-CreER^T2^ lineage-traced β2 tanycytes give rise almost exclusively to neurons in the median eminence in a diet-responsive manner (Lee et al., 2012). Later, during the early post-weaning period, Fgf10-expressing β tanycytes primarily generate orexigenic neurons in the arcuate nucleus, but also contribute neurons to the ventromedial, dorsomedial, and lateral hypothalamic nuclei, as well as produce a small number of parenchymal astrocytes (Haan et al., 2013). Most studies on neural stem potential in the mature hypothalamus, on the other hand, suggest that this capability resides with α tanycytes. *In vivo*, GLAST-expressing α tanycytes can self-renew and give rise to β1 tanycytes, while most of their parenchymal progeny are astrocytes rather than neurons (Robins et al., 2013). Additionally, a subset of α2 tanycytes characterized by *Prss56* expression are thought to generate neurons and astrocytes in the arcuate and dorsomedial nuclei, potentially *via* a transitory population of parenchymal tanycyte-like cells that translocate their soma from the third ventricle wall (Jourdon et al., 2016). The gliogenic potential of α tanycytes appears to decrease over time, however, as fewer newborn glia than neurons—presumed to be α tanycyte-derived based on proximity to the corresponding domain along the third ventricle—are observed in the mouse hypothalamus at 9 and 16 months of age (Chaker et al., 2016). Overall, diverse subpopulations of tanycytes possess neural stem potential, which may suggest the presence of distinct neurogenic and/or gliogenic zones along the third ventricle, but their lineage relationships, if any, are still unclear.

**FIGURE 2:**
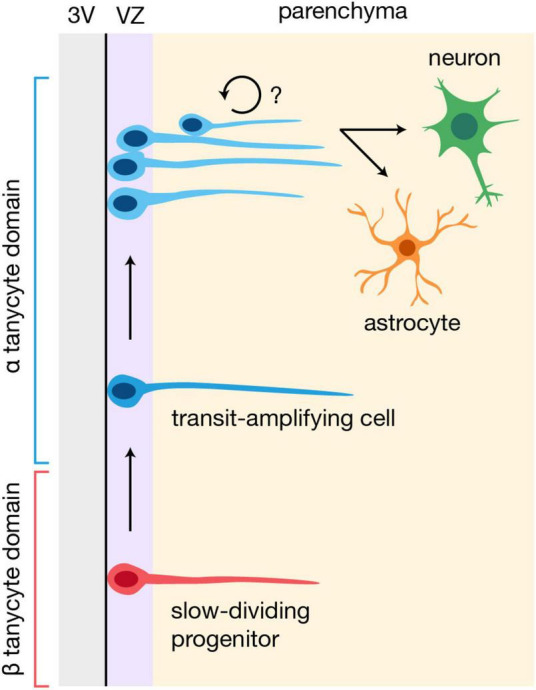
Potential model of neurogenesis and gliogenesis in the postnatal hypothalamus. β tanycytes may constitute slow-dividing progenitors that give rise to highly proliferative, transit-amplifying α tanycytes, which then generate cells that migrate into the hypothalamic parenchyma and differentiate into neurons or glia (Goodman et al., 2020; Yoo et al., 2021). It is unclear whether the parenchymal progeny of α tanycytes divide further before differentiating. 3V, third ventricle; VZ, ventricular zone.

At present, there is no consensus on the hierarchical organization of the hypothalamic niche. Some have speculated that α tanycytes constitute the bona fide neural stem cells and β tanycytes are more committed neuronal progenitors, given that α tanycytes can generate β1 tanycytes, and further, that only α tanycytes are neurospherogenic (Robins et al., 2013; Prevot et al., 2018). It is important to note, however, that β tanycytes constitute a relatively dormant, slow dividing stem-population under physiological conditions (Mu et al., 2021), and that the neurosphere assay does not reliably identify quiescent neural stem cells (Codega et al., 2014). Indeed, Fgf10-expressing β tanycytes are reported to incorporate BrdU at a slower rate than α tanycytes, and to give rise to highly proliferative α tanycytes (Goodman et al., 2020). These newly generated α tanycytes, which resemble transit-amplifying cells, can subsequently divide to generate daughter cells that migrate laterally into the hypothalamic parenchyma (Goodman et al., 2020), which may be related to the Prss56^+^ lineage parenchymal tanycyte-like cells noted above (Jourdon et al., 2016). While it is not yet clear whether these parenchymal descendants divide further before differentiating, or are at all related to the controversial parenchyma-residing progenitors that have been reported (Yoo and Blackshaw, 2018), this model thus places β tanycytes at the top of the hypothalamic neural stem cell hierarchy, followed by their transit-amplifying α tanycyte descendants (Figure 2; Goodman et al., 2020). Interestingly, RNA velocity analysis of a scRNA-seq dataset consisting of postnatal tanycytes and tanycyte-derived cells also indicates that neuronal precursors arise from α2 tanycytes that enter a proliferative state (Yoo et al., 2021). Finally, and perhaps unsurprisingly, ventricle-derived parenchymal progenitors have also been observed in both the developing mouse and human hypothalamus (Zhou et al., 2020), and regulators of postnatal tanycyte neurogenesis, such as Fgf signaling (Goodman et al., 2020) and the NFI family of transcription factors (Yoo et al., 2021), also modulate hypothalamic progenitor cells during development (Burbridge et al., 2016; Romanov et al., 2020). Taken altogether, this suggests that while the constituents of the hypothalamic niche may evolve over time—including a varying combination of radial glial cells, tanycytes, and potentially other neural stem populations (Son et al., 2021), at different developmental stages—the overall process of neurogenesis and gliogenesis in the region likely remains largely the same.

## Discussion

Tanycytes, which bear morphological, spatial, and molecular resemblance to embryonic radial glia, are unique in that they not only modulate and participate in various hypothalamic functions, but also remodel the corresponding neural circuits by supplying new neurons and glia. Notably, the developmental programs controlling tanycyte differentiation appear to be initiated relatively early in neurodevelopment, raising several interesting questions. For example, are tanycytes functional neural stem cells in the embryonic hypothalamus, and if so, do they contribute to developmental neurogenesis and/or gliogenesis alongside classical radial glia, potentially in an environmentally responsive manner? In the mature hypothalamus, tanycytes are a heterogeneous population of cells, and it is possible that subpopulations beyond the four classical subtypes—perhaps along the often-overlooked rostrocaudal axis—will be identified. This heterogeneity complicates efforts to understand the organization of the postnatal hypothalamic niche, but a growing body of evidence indicates that tanycyte-derived neurons and glia populate diverse regions of the hypothalamus. Notably, given the low basal levels of tanycyte-derived neurogenesis and gliogenesis, it may be necessary to challenge or perturb the system to determine the physiological relevance of this enigmatic niche across a range of developmental stages.

## Author contributions

HF prepared the manuscript and the figures, with the assistance of the DMK who helped edit. Both authors contributed to the article and approved the submitted version.
